# Pursuing precision in medicine and nutrition: the rise of electrochemical biosensing at the molecular level

**DOI:** 10.1007/s00216-023-04805-5

**Published:** 2023-07-07

**Authors:** Susana Campuzano, Rodrigo Barderas, Maria Teresa Moreno-Casbas, Ángeles Almeida, José M. Pingarrón

**Affiliations:** 1 Departamento de Química Analítica, Facultad de CC. Químicas, Universidad Complutense de Madrid, 28040 Madrid, Spain; 2 UFIEC, Instituto de Salud Carlos III, Majadahonda, 28220 Madrid, Spain; 3 Nursing and Healthcare Research Unit (Investén-isciii), Instituto de Salud Carlos III, Madrid, Spain; 4 Biomedical Research Center Network for Frailty and Healthy Ageing (CIBERFES), Instituto de Salud Carlos III, Madrid, Spain; 5 Instituto de Biología Funcional y Genómica, CSIC, Universidad de Salamanca, Salamanca, Spain; 6 Instituto de Investigación Biomédica de Salamanca, Hospital Universitario de Salamanca, CSIC, Universidad de Salamanca, Salamanca, Spain

**Keywords:** Precision medicine and therapy, Individualized nutrition, Electrochemical biosensing, Molecular markers

## Abstract

In the era that we seek personalization in material things, it is becoming increasingly clear that the individualized management of medicine and nutrition plays a key role in life expectancy and quality of life, allowing participation to some extent in our welfare and the use of societal resources in a rationale and equitable way. The implementation of precision medicine and nutrition are highly complex challenges which depend on the development of new technologies able to meet important requirements in terms of cost, simplicity, and versatility, and to determine both individually and simultaneously, almost in real time and with the required sensitivity and reliability, molecular markers of different omics levels in biofluids extracted, secreted (either naturally or stimulated), or circulating in the body. Relying on representative and pioneering examples, this review article critically discusses recent advances driving the position of electrochemical bioplatforms as one of the winning horses for the implementation of suitable tools for advanced diagnostics, therapy, and precision nutrition. In addition to a critical overview of the state of the art, including groundbreaking applications and challenges ahead, the article concludes with a personal vision of the imminent roadmap.

## Introduction

In these days when we seek individuality in everything: consumer products, movie services, music, etc., it is not surprising that the quest for personalization in medicine, therapy and nutrition would be more important than ever. But this is also a particularly relevant need, given the disturbing context in which we live, beginning to see the light of day after the COVID pandemic, and with Brexit, the Russian invasion of Ukraine and rising inflation.

There is increasingly strong evidence that if we advance in research, management, and implementation of precision medicine, therapy, and nutrition, we will assure a longer and higher quality of life, a more rational use of resources and, in addition, we will have at our disposal the right to be participants in our self-care [1].

According to recent trends, both research and implementation of precision medicine, therapy, and nutrition (understood not as the development of unique treatments or diets for each individual but the application of those available that best suit to the unique characteristics of each individual) involves both the identification of markers at different molecular levels and the development of disruptive technologies that allow their multidetermination and validation. It is widely accepted that the individual variability, the heterogeneity of certain diseases, and the absence of exclusive markers demand their multiplexed determination even at the multiomic level to improve the accuracy in the diagnosis, prognosis, and therapy. There are contexts that require developing a photograph with the highest possible resolution (maximum pixels) to obtain an accurate image of what is in front of us. The technologies pursued must also be affordable, easy to use, extremely versatile in design and application, sustainable, capable of analyzing human fluids both outside and in the body and ideally in real time, continuously, without the need for reagents or calibrations and in any environment.

In this context, the unprecedented evolution of electrochemical bioplatforms and instrumentation in recent years has positioned them as tools that come very close to being able to meet all these requirements.

Considering this state of the art, the purpose of this review article is to discuss, based on selected cutting-edge examples, the unique opportunities and advances that electrochemical biosensors offer to advance in sustainable and affordable precision medicine, therapy, and nutrition for everyone. Although some recent reviews and editorials of interest have echoed the potential of biosensors in precision therapy [2] and of wearable biosensors in precision medicine [3] and nutrition [4] to our knowledge none of them jointly discusses the potential of electrochemical biosensing in the three fields (medicine, therapy, and nutrition), which, moreover, we consider of particular relevance because of their indisputable interrelation. This critical review fills this important gap and concludes by highlighting, from a somewhat more personal perspective, the challenges and future prospects to be faced.

## Electrochemical bioplatforms: bringing precision to medicine and nutrition

Electrochemical bioplatforms, with a letter of introduction demonstrating high success for the determination of a wide range of molecular markers relevant in different fields, possess unique features comprising simplicity, high sensitivity and selectivity, cost-effectiveness, rapid response, ability to be highly customizable and adaptable, inherent miniaturization, scalable manufacturing, use of energy-efficient instrumentation, easy adaptation for wireless data transfer, implementation on different types of substrates, and integration into portable or indispensable (smart-phone/watches) devices [4]. The versatility they have allows providing point-of-care multi-omics biomarker profiling on complex and poorly treated samples, requiring shorter assay times and smaller sample quantities than other leading technologies available for centralized determination of proteomic, glycomics, transcriptomic, and epigenetic biomarkers [5]. It is important to note that some developments have shown potential to provide real-time information in a single-step, high frequency, reversible, reagentless, calibration-free way, and to operate continuously for several hours in complex raw matrices both in flow and *in vivo* [6]. All these singularities position electrochemical bioplatforms as suitable alternatives to address the challenges posed today by research and implementation of precision medicine, therapy, nutrition, and agriculture [7].

The unprecedented advances that bioelectroanalytical methods have undergone in recent years and that, as will be seen in the following sections, have allowed bringing precision to our lives, have been assisted, among other things, by:

i) Advances in the design and fabrication of electrochemical substrates in terms of use (reusable or disposable screen-printed electrodes [8–10]), fabrication materials (paper, food, or ingestible products [11], plastic, textile, polymeric) and properties (superwettable [12], flexible and stretchable [13]) and the progress in miniaturized bioelectrochemical electronics [14, 15] that have combined seamlessly into new electrochemical sensor formats: wearables [3, 16–19], implantable [20], microneedle-based [21], etc.
ii) The production and application of new bioreceptors including biomolecular switches [6, 22], natural cell membranes [23] or those obtained through innovative technologies (HaloTag [24–26], phage-display [27] and targeted mutation [28]), overcoming the limited commercial availability or the problems that conventional strategies for their production/purification/storage may present, and even allowing the discrimination of viral variants.
iii) The development of new strategies to correct the baseline drift experienced by electrochemical responses *in vivo* [29–34] and to develop calibration-free electrochemical biodevices [35].
iv) The preparation of surfaces with antibiofouling properties that allow both direct determination and continuous operation in undiluted biofluids [36–38] by exploiting the use of Self Assembled Monolayers (SAMs) that mimic the surface of cell membranes [29, 35], directly cell membranes [23], or transient polymeric coatings [39].

It is this empowerment in properties and opportunities that has allowed electroanalytical bioplatforms to make inroads and demonstrate pioneering applications to assist in precision medicine, therapy, and nutrition. The following sections critically discuss the rationale and remarkable merits of the breakthroughs that have come to light in the last years inviting us to believe that precision in medicine and nutrition are achievable by electrochemical biosensing of molecular markers.

## Precision medicine: diagnosis, monitoring, and therapy

Although "precision medicine" and "personalized medicine" are considered interchangeable terms differing only in the moment they emerge intime, with the aim of the term "personalized"does not mislead in thinking about the development of unique treatments for each individual, it is now considered more appropriate to speak of precision medicine or PM.

While PM is not a new concept, in recent years it has gained more importance and momentum supported with the help of world leaders such as the former U.S. President Barack Obama, who in early 2015 announced the "Precision Medicine Initiative", with a mission to "bring us closer to curing diseases like cancer and diabetes, and give us all access to the personalized information we need to keep us and our families healthy" [20].

Thus, PM is defined as the prevention, investigation and treatment of diseases taking into account individual variability [40, 41]. This personal fingerprint is considered to include genetic and molecular biomarkers (preferably present in samples that can be taken frequently and minimally invasive that reflect biological alterations with prognostic or predictive value), phenotype, environment, and lifestyle.

Therefore, this approach should make it possible to classify individual patients into subpopulations differing in their susceptibility to a given disease, prognosis and response to treatment. This classification seeks to adapt treatments (in terms of type, dose and timing of application) to the attributes and/or stage of the disease of each individual, thus improving their efficacy and minimizing unnecessary adverse effects.

It is also important to emphasize that the fact that a patient can access to this type of medicine is a right that makes him/her aware of his/her self-care and enjoy a longer and better quality of life and, in the eyes of society, implies a more rational and economical use of resources.

The importance of PM, more established so far in oncologic diseases, is increasing also in other types of diseases involving genetic abnormalities and individual biological particularities such as neurodegenerative and infectious diseases. However, although PM is already leading to higher survival rates in cancer patients with worse prognosis, its implementation in egalitarian healthcare is conditioned by the deployment of innovative, affordable technologies that allow the identification, validation and determination of new biomarkers, as well as novel tools (informatics, computational and artificial intelligence) enabling precise patterns and profiles of each individual and their disease, and by the approval of ethical and regulatory issues.

The advances demonstrated in recent years by electrochemical bioplatforms for the simple, rapid, and reliable determination of molecular markers of accepted clinical relevance in different biological fluids and for the identification of new molecular signatures suggest that these biodevices undoubtedly have much to say and contribute both in research and in the implementation of precision medicine, encompassing the diagnosis, prognosis, and follow-up of diseases. Table 1 summarizes relevant characteristics of a representative sample of the recent developments expressly aimed to contribute to precision medicine through the determination of target biomarkers.

**Table 1:** Representative examples of electrochemical bioplatforms for interrogating precision medicine-related molecular markers in body fluids

Electrode	Rationale	Target biomarker/Target disease	Electrochemical transduction	Analytical characteristics	Other remarkable features	Application	Ref.
Carbon electrodes printed on PET	Enzymatic (HBD) electrochemical test strip	HB/DK and DKA	Chronoamperometry (Fe(II)/NADH)	LR: 0.001−6.100 mM LOD: 0.001 mM	Medical dongle powered by a smartphone	Blood from DKA patient	[42]
CPEs	Multi-electrochemical biosensor (GOx-PB electrodes) array with controlled delayed sensing capability achieved by modifying the electrode surfaces with transient methacrylate polymeric coatings	Glucose/--	Chronoamperometry (PB/H_2_O_2_)	--	Antibiofouling properties	Continuous operation in in undiluted saliva and blood samples (up to 6 h)	[39]
CPEs (edible materials)	CPEs (edible paste, GOx and olive oil) modified with transient methacrylate polymeric coatings	Glucose	Chronoamperometry (PB/H_2_O_2_)	LOD < 1 mM	Antibiofouling properties, preservation of enzymatic activity in extreme pH values	Continuous operation in GI fluids	[43]
AuEs	Dual-marker biosensor chip, integrating enzymatic (GOx) and HRP-labeled sandwich immunoassay for glucose and insulin, respectively	Glucose and insulin/DM	Chronoamperometry (Glucose: TTF/H_2_O_2_; insulin: TMB/H_2_O_2_)	Glucose: LR: 2–18 mM LOD: 209 µM Insulin: LR: 0.5–3 nM LOD: 340 pM	Disposable, dual determination in a single microliter sample droplet within less than 30 min	Untreated whole blood and raw saliva samples	[44]
AuE	Switch-based aptamer labeled with MB	Phenylalanine/Phenylketonuria	SWV (MB)	LR: 90 nM−7 μM	Calibration-free	Unprocessed, finger-prick volumes of whole blood Rapid: < 10 min	[45]
Pt-NC working flexible electrode	Pt nano-cluster/enzyme (ChOx) /Nafion	Cholesterol/ Cardiovascular diseases	Amperometry (H_2_O_2_)	LR: 2−486 µM LOD: 2 µM	Disposable, flexible	Saliva from patients with hyperlipidemia	[46]
AuEs	Dual electrochemical immunosensor microchip by integrating different enzymatically-tagged competitive (cortisol, ALP) and sandwich (insulin, HRP) immunoassay formats on a single chip platform	Cortisol and insulin/DM	Chronoamperometry (Cortisol: 1-NPP; insulin: TMB/H_2_O_2_)	Cortisol: LR: 0–250 ng mL^−1^ LOD: 13.4 ng mL^−1^ Insulin: LR: 0−200 pM LOD: 30.6 pM	Disposable, dual determination in a single microliter sample droplet within less than 25 min	Untreated serum samples	[47]
ePAD (SPGE)	Direct immunosensor and Ab covalently immobilized on the GO/SPGE	Ferritin/Anemia	DPV ([Fe(CN)_6_]^3−/4−^)	LR: 1−1000 ng mL^−1^ LOD: 0.19 ng mL^−1^	Paper-based, handmade fabricated, disposable, label-free, 3 weeks storage stability	Spiked serum samples	[48]
GEs	Immunoassays implemented on a laser-engraved G multiplexed platform	SARS-CoV-2 N protein, N-specific IgG and IgM, and CRP/SARS-CoV-2	DPV (HQ/H_2_O_2_)	--	Wireless, multiplexed, rapid, individualized snapshot of COVID-19 infection	Serum and saliva samples from SARS-CoV-2 infected individuals	[49]
Wearable tattoo PB electrode	Wearable platform integrating a screen-printed iontophoretic electrode system for ISF extraction by RI, a printed three electrode amperometric glucose biosensor, and an electronic interface for control and wireless communication	Glucose/DM	Chronoamperometry (PB/H_2_O_2_)	LR: 0−22 mM	Skin-worn, disposable, flexible, wireless, prolonged on-body glucose monitoring of up to 8 h	Clinical evaluations of the sensor in ISF from DM patients for 4 h and prolonged on-body operation (up to 8 h)	[50]
PB SPE	Simple touch-based fingertip sweat electrochemical enzymatic (GOx) biosensor new algorithm that addresses for personal variations	Glucose/DM	Chronoamperometry (PB/H_2_O_2_)	--	Painless, no sweat stimulation, simple one-time personal precalibration, personalized sweat-response-to-blood concentration translation	Sweat from the fingerprint	[51]
PB SPE modified with GOx	Epidermal patch for the simultaneous monitoring of haemodynamic and metabolic biomarkers	Glucose, lactate, alcohol, caffeine, BP and HR/--	Chronoamperometry (PB/H_2_O_2_)	--	Wearable, Multimodal sensors that fuse acoustic and electrochemical sensors	*On body* testing of glucose, lactate, alcohol, and BP in healthy consenting individuals (sweat and ISF)	[52]
SPE modified with GOx, PB and NiHCF	Integration of an electrochemical biosensor with HMNs arrays for ISF extraction	Glucose/DM	Chronoamperometry (PB/H_2_O_2_)	LR: 2.5 −15 mM	--	*In vitro* and ex vivo assays using porcine skin	[53]
PMMA-based microneedle microelectrodes ere fabricated using a micromachining method	Immobilization of the corresponding oxidase (AOx, LOx, GOx) mixed with chitosan between PPD and PVC layers	Lactate and glucose, or alcohol and glucose	Amperometry (H_2_O_2_)	Glucose: DR: 0–40 mM LOD: 0.32 mM Lactate: DR: 0–28 mM LOD: 0.15 mM Alcohol: DR: 0–100 mM LOD: 0.50 mM	Dual sensing, Wearable, wireless, continuous real-time sensing, coupled with a custom smartphone app for data capture and visualization, 12 h of continuous operation	*On body* operation (ISF)	[54]
Au-coated SPCE modified with TBO, HBD, NAD^+^, CNTs and Chit	Enzymatic (HBD) electrochemical test strip	HB/--	Chronoamperometry (TBO/NADH)	LR: 0.1−3.0 mM LOD: 50 μM (in an artificial saliva medium)	Coupled with a hand-held electrochemical analyzer near real-time Salivary HB detection to track non-invasively the dynamics of HB concentrations after intaking commercial supplements	Human saliva samples collected from healthy volunteers	[55]
AuE	Pseudoknot-assisted electrochemical aptasensor integrated with microfluidic sweat bioelectronic patch in a wearable format	Cortisol/ Chronic stress	DPV (MB)	LR: 1 pM–1 µM LOD: 0.2 pM	Wearable, label-free, continuous monitoring up to 90 min	*On body* evaluation in healthy individuals (sweat)	[56]
SCPEs	Indirect immunoassays implemented at MBs modified with HaloTag-peptides	AAbs against 6 specific HaloTagged peptides: 4 phage display and 2 aberrant/AD	Amperometry (HQ/H_2_O_2_)	--	Full AD-diagnostic capability, POC multiplexing platform to detect the signature in a single test (1 h 15 min)	Serum samples from AD patients	[27]
SCPEs	Indirect immunoassays implemented at MBs modified with HaloTag-TAAs	AAbs against 8 CRC-specific circulating HaloTagged-TAAs/CRC	Amperometry (HQ/H_2_O_2_)	--	Successful discrimination between CRC and premalignant subjects from control individuals with great specificity and sensitivity	Serum samples from patients with breast and lung cancers, CRC, and premalignant CRC lesions	[24]
SPCEs	Indirect immunoassays implemented at MBs modified with HaloTag-TAAs	AAbs against 15 CRC-specific exosomal HaloTagged-TAAs/CRC	Amperometry (HQ/H_2_O_2_)	--	Successful discrimination between CRC and premalignant subjects from control individuals with great specificity and sensitivity	Serum samples from CRC patients and individuals with premalignant CRC lesions	[25]
SPCEs	Indirect immunoassays implemented at MBs modified with N protein and ectodomains of S protein produced by directed mutation	N- and S-specific IgGs, IgMs and IgAs/SARS-CoV-2	Amperometry (HQ/H_2_O_2_)	--	Identification of vulnerable populations from those with natural or acquired immunity, monitoring of infection, evaluation of vaccine efficiency, identification of the variant responsible for the infection	Serum samples from SARS-CoV-2 (Wuhan variant) infected patients	[28]
SPCEs	Indirect immunoassays implemented at MBs modified with p53 and p63 HaloTag-proteoforms	AAbs against specific p53 and p63 proteoforms/CRC	Amperometry (HQ/H_2_O_2_)	--	Successful discrimination between CRC and premalignant subjects from control individuals with great specificity and sensitivity	Serum samples from CRC patients and individuals with premalignant CRC lesions	[26]

AAbs: autoantibodies; AD: Alzheimer's disease; AOx: alcohol oxidase; BP: blood pressure; Chit: chitosan; ChOx: cholesterol oxidase; CNTs: carbon nanotubes; CPEs: carbon paste electrodes; CRC: colorrectal cancer; CRP: C-reactive protein; DK: diabetic ketoacidosis; DKA: diabetic ketosis acid; DM: diabetes mellitus; ePAD: electrochemical paper-based analytical device; GEs: graphene electrodes; GI: gastrointestinal; GO: graphene oxide; GOx: glucose oxidase; HB: β-hydroxybutyrate; HBD: β-hydroxybutyrate dehydrogenase; HMN: hollow microneedle; HQ: hydroquinone; HR: heart rate; HRP: horseradish peroxidase; ISF: interstitial fluid; LOx: lactate oxidase; LR: linear range; LOD: limit of detection; MBs: magnetic beads; MB: methylene blue; N: nucleocapsid protein; NAD^+^: nicotinamide adenine dinucleotide; NiHCF: nickel hexacyanoferrate; 1-NPP: 1-naphthyl phosphate; PB: prussian blue; PET: polyethylene terephthalate; PMMA: poly(methyl methacrylate); PPD: poly-o-phenylenediamine; Pt-NC; platinum nano-cluster; PVC: polyvinyl chloride; RI: reverse iontophoresis; S: spike protein; SPCEs: screen-printed carbon electrodes; SPGE screen-printed graphene electrode; TAA: tumor-associated antigen; TBO: toluidine blue O; TMB: 3,3´,5,5´-tetramethylbencidine; TTF: tetrahiafulvalene

As can be deduced from the information in Table 1, bioplatforms constructed in disposable, paper-based, flexible and wearable formats have been reported for the rapid and reliable single or simultaneous determination of biomolecules, such as β-hydroxybutyrate (HB), cholesterol, glucose, insulin, phenylalanine, cortisol, and ferritin which are of clinical relevance in prevalent diseases such as diabetes mellitus (DM), diabetic ketoacidosis (DK), diabetic ketosis acid (DKA), phenylketonuria, anemia, and cardiovascular diseases. To achieve the required selectivity many of the strategies employ catalytic bioreceptors while only a few use affinity bioreceptors such as antibodies [44, 47] and aptamers [45, 56]. It is important to highlight the use of aptamer switches [45], which belong to the group of the so-called biomolecular switches [45], exploited in the development of reagentless self-generating signal electrochemical biosensors, based on altering the efficiency with which the redox reporter approaches the electrode surface upon binding of the recognition element to the target analyte [57, 58]. Table 1 also shows that many bioplatforms have been applied to the direct determination of biomarkers in sparingly treated extracted biofluids (blood, saliva, serum, and tears), and some biodevices developed in wearable formats have been applied for *on-body* analysis of interstitial fluid (ISF) and sweat, using iontophoretic systems [50], microneedle-based formats [53], or taking advantage of the fast sweat rate on the fingertip [51]. Electrochemical transduction is mostly performed by amperometry but voltammetry [44, 48, 49, 56] and label-free approaches [48, 56] have also been used.

Importantly, the developed bioplatforms provide analytical characteristics compatible with their clinical applicability and have demonstrated to be useful for the analysis of blood and saliva samples of patients with DKA [42] and hyperlipidemia [46] and ISF of patients with DM [50] in research settings.

It is noteworthy that the developed bioplatforms allow operating in biofluids [39] or *on-body* [50] for prolonged times. This ability relies, for example, on the use of transient methacrylate-based coatings, with different dissolution times at specific pH values, leading to delayed exposure of the fresh transducer surface [39]. Indeed, the excellent protective properties imparted by these temporary coatings were exploited to ensure the biocatalytic activity of edible enzymatic electrochemical biosensors (manufactured with food or ingestible products) in media with extreme pH values such as gastrointestinal fluids [43].

**Fig. 1:**
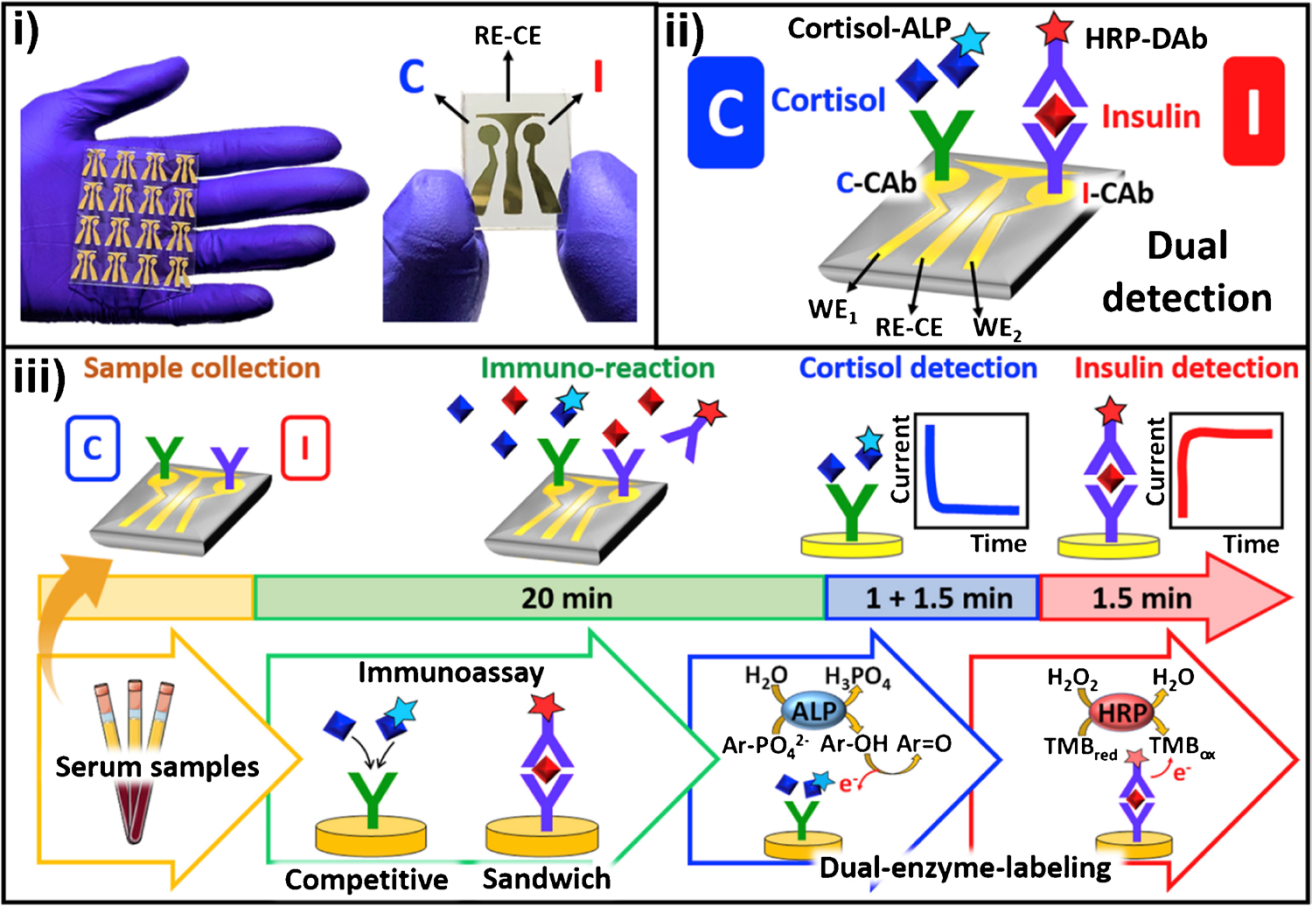
Dual immunochip for the determination of cortisol I and insulin (I): i) Actual image of the chips using a three-electrode Au sputtered system; **ii**) Schematic of the implemented immunoassays involving different formats and enzymatic tracers (competitive/ALP for C and sandwich/HRP for I); and **iii**) Immunoreaction and transduction processes monitored by chronoamperometry (+0.4 V in the presence of 1-naphthyl phosphate (1-NPP) for C and −0.1 V in the presence of 3,3´,5,5´-tetramethylbencidine (TMB)/H_2_O_2_ for I). Reproduced from [47] with permission

**Fig. 2:**
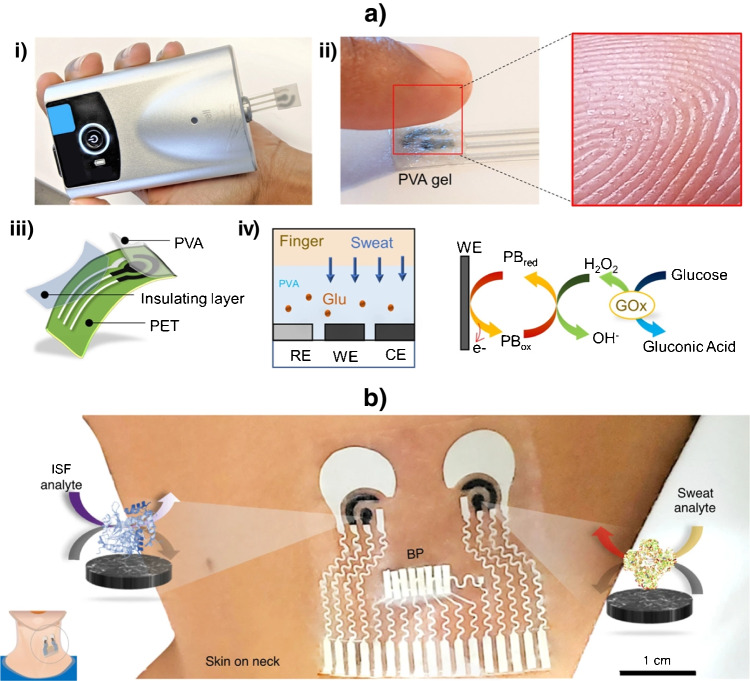
**a**) Bloodless fingerstick sweat glucose biosensor: i) Portable potentiostat attached to the biosensor; ii) image of the biosensor and fingertip showing the sweat glands; iii) Schematic of the tactile biosensor showing the substrate (polyethylene terephthalate, PET), the screen-printed sensor, and the insulating and polyvinyl alcohol (PVA) layers; and iv) schematic showing the collection of sweat from the fingertip, through the PVA layer, to the working electrode and schematic enzymatic and electrochemical reactions occurring at the electrode. **b**) Multimodal epidermal patch that combines acoustic and electrochemical enzymatic biosensors for *on body* simultaneous testing of hemodynamic and metabolic biomarkers. Illustrations of sensor placement for blood pressure (BP) and enzymatic chemical sensors for ISF and sweat. Reproduced from a) [51] and b) [52] with permission

**Fig. 3:**
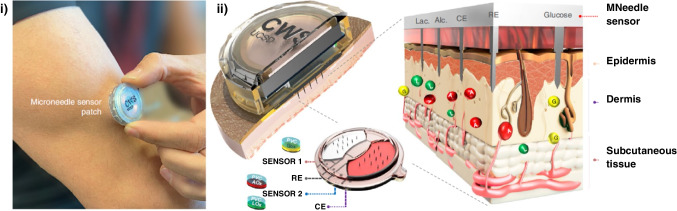
Wearable integrated microneedle array for the simultaneous *on body* monitoring of lactate and glucose, or alcohol and glucose in ISF: **i**) Sensor patch attached to a user’s arm; and **ii**) Cross-sectional illustration of the sensor patch with the microneedles piercing the skin to reach the epidermis, along with the configuration of the microelectrodes on the multiplexed sensor component and scale-free image of the close-up cross-section of the microneedle/skin interface. Reproduced from [54] with permission

Due to their relevance and novelty, the disposable biosensor chips reported by Wang’s team (at UCSD) for double determination of cortisol and insulin in a single microliter of untreated samples (whole blood, saliva, and serum) in less than 30 minutes combining in the same platform catalytic and affinity bioassays [44] or immunoassays based on different formats (sandwich and competitive) and enzymatic tracers (alkaline phosphatase, ALP, and horseradish peroxidase, HRP) (Fig. 1) [47], deserve special attention. The same team prepared a simple touch-based fingertip sweat electrochemical enzymatic biosensor to track glucose in combination with a new algorithm that addresses for personal variations and does not require sweat stimulation. The biosensor allowed simple one-time personal pre-calibration and translation of sweat response to blood concentration (Fig. 2a) [51] and the preparation of the first multimodal epidermal patch combining acoustic and electrochemical sensors for the *on body* simultaneous testing of hemodynamic (blood pressure, BP, and heart rate, HR) and metabolic (glucose, lactate, alcohol, and caffeine) biomarkers (Fig. 2b) [52]. The same group participated in the development of a wearable integrated microneedle array coupled with a custom smartphone app for data capture and visualization for the simultaneous *on body* monitoring of lactate and glucose, or alcohol and glucose in ISF (Fig. 3) [54].

Also noteworthy is the non-invasive wearable stress patch reported by Singh et al. [56] for real-time and continuous (up to 90 min) cortisol monitoring using a pseudoknot-assisted aptamer.

Although the COVID-19 pandemic experienced at global level gave rise to a huge boom of promising electroanalytical biotools for assisting in its detection and control, it is worth highlighting the multiplexed bioplatforms reported by Gao's team [49] and by the Pingarrón/Campuzano and Barderas teams [28] for the management of SARS-CoV-2 infection.

**Fig. 4:**
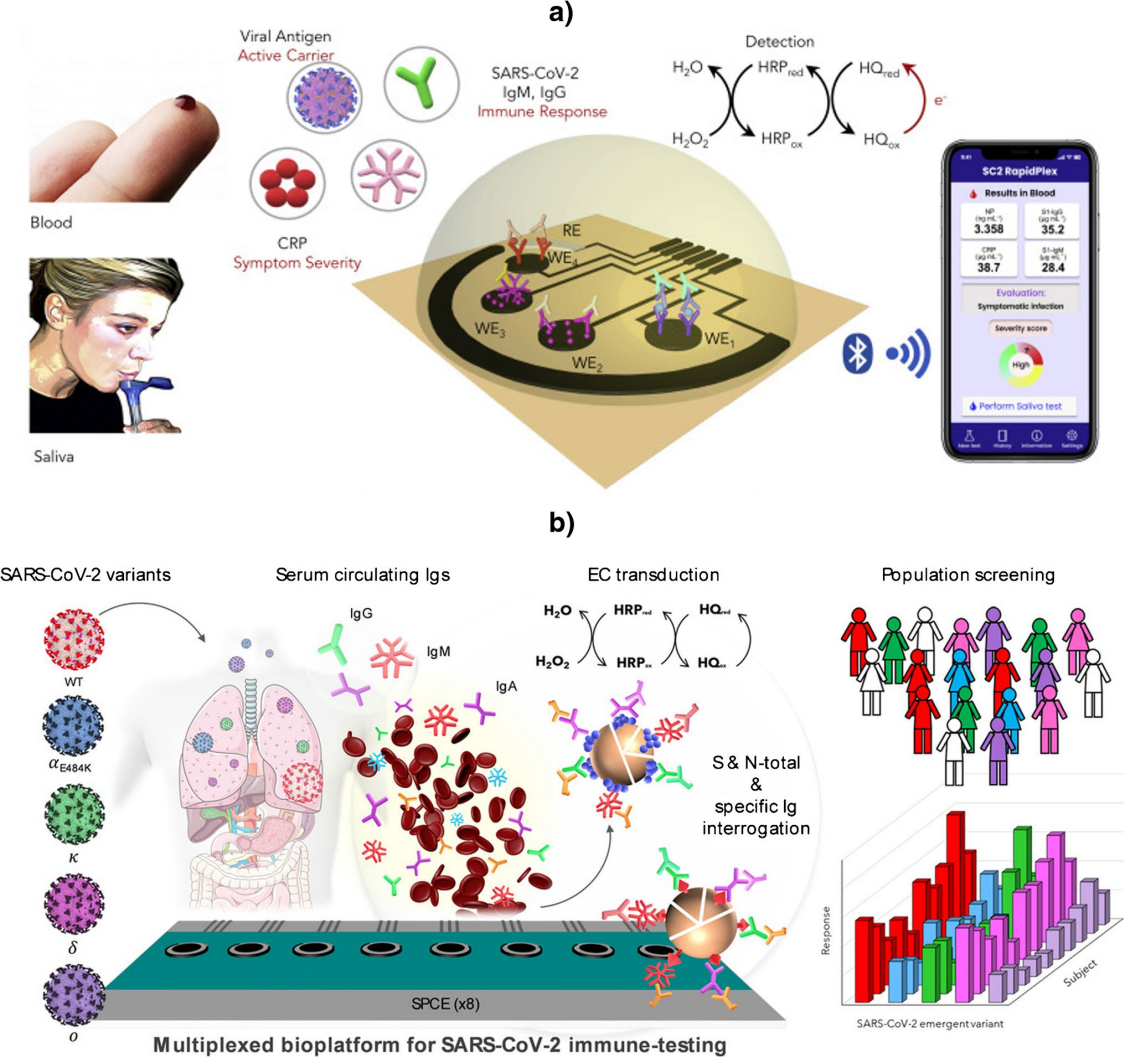
**a**) Wireless platform using disposable and flexible laser-engraved graphene arrays for the rapid and multiplexed electrochemical detection of SARS-CoV-2 nucleocapsid (N) protein, the antibodies generated by the immune system against N protein (N-specific IgGs and IgMs) and CRP. **b**) Multiplexed amperometric bioplatform able to quantify total and isotype N- and spike (S)-specific anti-SARS-CoV-2 serum immunoglobulins (Igs) and to analyze the global and isotype-specific immune response of COVID-19 convalescent and vaccinated individuals, as well as to detect neutralizing antibodies specific to protein S of variants of concern. Reproduced from a) [49] and b) [28] with permission

Gao's group proposed a fast, wireless, remote, high sampling frequency, multiplexed telemedicine platform involving immunoassays performed at low-cost laser-engraved graphene electrodes, capable of detecting with the same device three key aspects of the disease viral load (nucleocapsid, N, protein, as a viral carrier), the immune response (IgM and IgG antibodies against the S1 virus) and the severity of the disease (inflammatory biomarker CRP). The approach demonstrated its reliability for the analysis of blood and saliva samples from infected and uninfected individuals (Fig. 4a) [49]. The other highlighted bioplatform for the detection of SARS-CoV-2 [28] implemented competitive immunoassays on the surface of magnetic microparticles (MBs) modified with commercial N protein or ectodomains of spike (S) protein produced by targeted mutation and was applied to the determination of N- and S-specific IgGs, IgMs and IgAs in serum (Fig. 4b). This SARS-CoV-2 electroanalytical biotool was able to: (i) reliably discriminate in 75 min infected from non-infected patients using 1000-fold diluted sera; (i“) "quantify" natural and/or acquired immunity after infection and/or vaccination processes, which allows both to evaluate the effectiveness of vaccination programs and to implement individualized vaccination strategies in time and dose; (iii) evaluate the humoral immune response against any variant that may emerge by in-house expressing its S ectodomain; and (iv) identify the variant responsible for the infection.

**Fig. 5:**
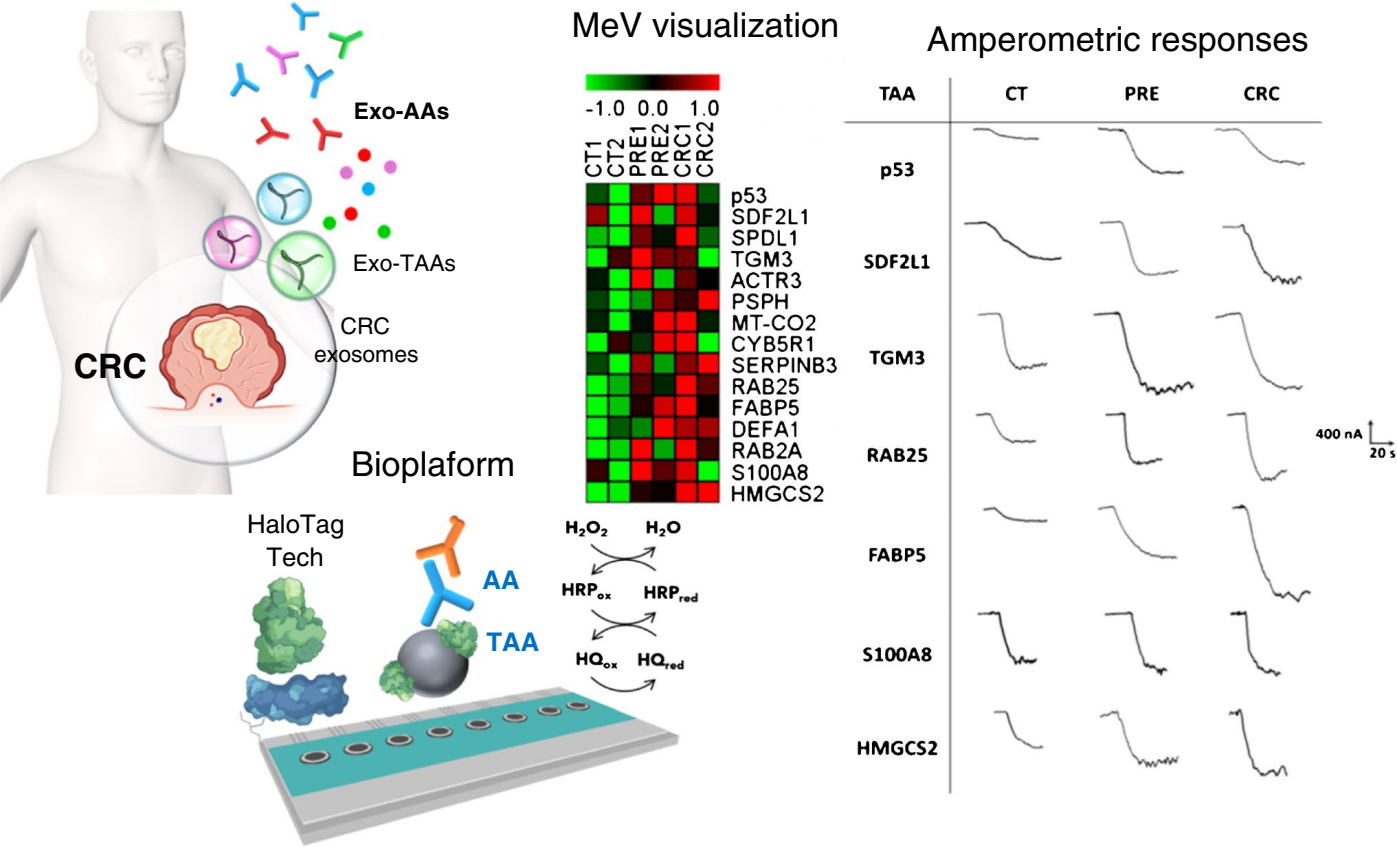
Multiplexed biosensing platforms to identify a novel signature comprising fifteen AAbs against tumor-associated antigens from exosomes released by colorectal cancer (CRC) cells and tissue samples with high diagnostic ability to discriminate healthy individuals from CRC patients and individuals with premalignant CRC lesions. Figure drawn by authors

The identification of new molecular signatures is considered essential to advance in the precision medicine for prevalent diseases. In this context, the work carried out by the group of Barderas and Pingarrón/Campuzano reported MBs modified with antigens of different nature identified by proteomics (circulating antigens, exosomal, peptides and proteoforms) and produced by HaloTag [59, 60] and phage display [61] technologies, which were coupled to disposable multiplexed electrochemical platforms, for the determination of the corresponding serum autoantibodies (AAbs). The application of these bioplatforms to the analysis of cohorts of healthy individuals and patients diagnosed with cancer and Alzheimer disease (AD) showed the usefulness of new characteristic signatures comprising AAbs against eight circulating tumor antigens [24], fifteen exosomal tumor antigens (Fig. 5) [25], or four proteoforms of p53 and p63 proteins [26], for the early diagnosis of patients with CRC and premalignant lesions of this neoplasm, as well as the signature involving AAbs against six peptides (four phage-deployed and two aberrant) for preclinical identification of AD [27].

One of the important concepts of PM is to select ‘the right drug for the right patient at the right dose and time’, what is known as precision therapy [2, 32]. Indeed, inappropriate drug dosing is another challenging issue facing the society and is one of the main causes of complications in antibiotic treatment. For example, more than 40,000 acute kidney injuries per year are reported because of tobramycin and vancomycin administration, with a treatment cost of more than 5 billion dollars per year in the United States [62].

The first step toward individualized pharmacotherapy is therapeutic drug monitoring (TDM), i.e., the clinical measurement of medication in a human body fluid (e.g., blood, saliva, or urine) at certain time intervals during treatment [2].

Recent studies have estimated that improvements in the accuracy of TDM methods is particularly relevant in the case of the most severely ill patients, as they are more prone to present metabolic alterations and have the narrowest therapeutic margin [31]. Such TDM accuracy can reduce these adverse outcomes by threefold. Nowadays, antibiotics are dosed according to the patient’s body weight without considering the high inter/intrasubject variations (the way drugs are absorbed and eliminated in the body and how the body responds to drugs) [31, 34, 62–64]. Because of these individualized dose-response characteristics of drugs, precision therapy is recently considered a new paradigm aimed at personalizing dosing to increase therapeutic efficacy and minimal toxic effects [64]. Indeed, it is well known that the success of antibiotherapy, crucial for patient safety and recovery, is largely dependent on the ability to maintain blood antibiotic concentrations within a safe therapeutic range tailored to the patient’s unique pharmacokinetic/pharmacodynamic response [65]. This is compounded because the analytical methods used to monitor patient-specific pharmacokinetics, based on blood draws followed by subsequent laboratory analysis, involve invasive/resource-intensive, and high-cost procedures that require hours or days to return pharmacokinetic estimates based on only one or two plasma drug measurements and, therefore, inadequate to allow timely intervention [31, 34, 62, 66].

Therefore, the development of new reliable, rapid, and inexpensive tools that can aid precision therapy by monitoring patients' personal pharmacokinetics at the bedside would facilitate the access to less toxic drug regimens with a higher probability of efficacy, the selection of alternative agents to antibiotics (thus minimizing antimicrobial resistance) and to ensure the safe and efficient delivery of drugs characterized by narrow therapeutic windows (range of plasma concentrations over which a molecule is therapeutically effective without causing significant adverse effects) or with highly complex and time-varying optimal plasma cycles to the most critically ill patients. All this would lead to major improvements in the quality of life of large groups of patients, minimize the side effects of drugs and reduce patient recovery times, healthcare costs and the prevalence of multidrug resistance [31, 34, 64].

Electrochemical bioplatforms have showed important advances in this direction, allowing continuous, real-time monitoring of blood drug levels, and providing an unprecedented avenue for improved TDM and, more generally, personalized, high-precision delivery of pharmacological interventions [31, 64]. Table 2 summarizes relevant features of the electrochemical bioplatforms reported for assisting precision therapy by tracking drugs in body fluids.

**Table 2:** Representative examples of electrochemical bioplatforms for assisting precision therapy by tracking drugs in body fluids and tissues

Electrode	Rationale	Target biomarker/Target disease	Electrochemical transduction	Analytical characteristics	Other remarkable features claimed by the authors	**Application**	**Ref.**
Dendritic gold coated, electrode on a silicon-based 16-channel neural recording probe substrate	Switch-based aptamer labeled with MB	Cocaine	SWV (MB)	--	Novel background subtraction and electrode calibration techniques allow for the removal of baseline drift artifact	*In vivo* response implanted in rat brain	[67]
AuE	Switch-based aptamer labeled with MB attached to a PC-terminated SAM	Doxorubicin or kanamicin	SWV (MB)	--	Minimization of baseline drift operating in undiluted whole blood, single-step, real-time, *in vivo* application	Flowing whole blood and jugular vein of live rats	[29]
PtE	Direct competitive bioassay exploing a naturally occurring PBP implemented in a disposable microfluidic chip	ß-lactam antibiotics: penicillins (piperacillin) and cephalosporins (cefuroxime and cefazolin)	Amperometry (H_2_O_2_)	LODs: 2.07, 4.88 and 5.71 ng mL^−1^ for piperacillin, cefuroxime and cefazolin	Disposable, microfluidic platform, detecting very low antibiotic concentrations (less than 6 ng mL^−1^) from only 1 µL of serum	Untreated plasma samples from patients	[63]
Gold wire electrode	Switch-based aptamer labeled with MB	Doxorubicin, kanamycin, gentamicin, tobramycin	SWV (MB) with KDM drift correction	--	Real-time, continuous, multihour measurements	Jugular vein of live rats	[30]
Gold wire electrode	Switch-based aptamer labeled with MB	Tobramycin	SWV (MB)	--	Closed-loop, feedback controlled delivery system	Jugular vein of live rats	[68]
Gold wire electrode	Switch-based aptamer labeled with MB	Tobramycin	Chronoamperometry		Calibration-free, resistant to drift	jugular vein of live rats	[69]
AuE	Switch-based aptamer dually labeled with MB and AQ attached to a PC-terminated SAM	Cocaine, ATP, kanamycin	SWV (MB, AQ)	--	Dual-reporter approach, calibration-free, single-step, real-time, *in vivo* application	Undiluted whole blood and jugular vein of live rats	[35]
Gold wire electrode	Switch-based aptamer labeled with MB	Vancomycin	SWV (MB) with KDM drift correction	--	Calibration-free, single-step, real-time, *in vivo* application	Finger-prick-scale samples of whole blood and jugular vein of live rats	[31]
AuE	Switch-based aptamer labeled with MB	Tobramycin	SWV (MB)	--	*In vivo* application	Jugular vein of live rats	[66]
Gold wire electrode	Switch-based aptamer labeled with MB	Irinotecan	SWV (MB) with KDM drift correction	--	Measurement of plasma irinotecan levels with micromolar concentration resolution and seconds temporal resolution high frequency, real-time	Jugular vein of live rats	[32]
PtE	Microfluidic biosensor based on Direct competitive PBP-based assay	Piperacillin	Amperometry (H_2_O_2_)	LOD: 0.058 μM	Finger-prick testing	Unprocessed human blood samples	[65]
PtE	Multiplexed microfluidic biosensor which combines CRISPR-powered and direct competitive PBP-based assay	COVID-19-specific RNA\ sequences and ß-lactam antibiotic (piperacillin/tazobactam)	Amperometry (H_2_O_2_)	LODs of 2,000 and 7,520 copies mL^−1^ for the *E* and *RdRP* genes	Multiplexing capacity (up to six analytes), miniaturized measurement setup, Sample-to-result time: ~30 min	Nasal swabs and serum samples of infected patients	[70]
AuNP-microneedle electrode	Microneedle-based electrochemical biosensing patch based on MB-labeled aptamers	Tobramycin and vancomycin	SWV (MB)	--	Continuous, real-time, low-cost fabrication scheme, strong correlation between the ISF/circulating drug levels	Artificial ISF buffer solutions and rat model	[62]
Au-coated microneedle electrode arrays	Microneedle Aptamer-Based Sensors	Irinotecan	SWV (MB) with KDM drift correction	--	Regenerable, *in vivo* application	ISF of a rodent	[33]
Gold wire electrode	Switch-based aptamer labeled with MB	Methotrexate	SWV (MB) with KDM drift correction	--	Real-time, *in vivo* application	Jugular vein of live rats	[34]

AQ: anthraquinone; DOX: doxorubicin; FTN: fentanyl; ISF: interstitial fluid; KDM: kinetic differential measurements; MB: methylene blue; PC: phosphatidylcholine; PBP: penicillin-binding protein; PPF: Propofol; SAM: self-assembled monolayer; SWV: square wave voltammetry

As it can be seen, two types of bioreceptors, aptamer switches and penicillin binding proteins (PBPs), have been used to meet the stringent demands of these applications. The aptamers have been used for the selective determination of irinotecan, vancomycin, tobramycin, doxorubicin, kanamicin, gentamicin, methotrexate, or cocaine and the bacterial proteins for the determination of ß-lactam antibiotics including penicillins (piperacillin) and cephalosporins (cefuroxime and cefazolin).

**Fig. 6:**
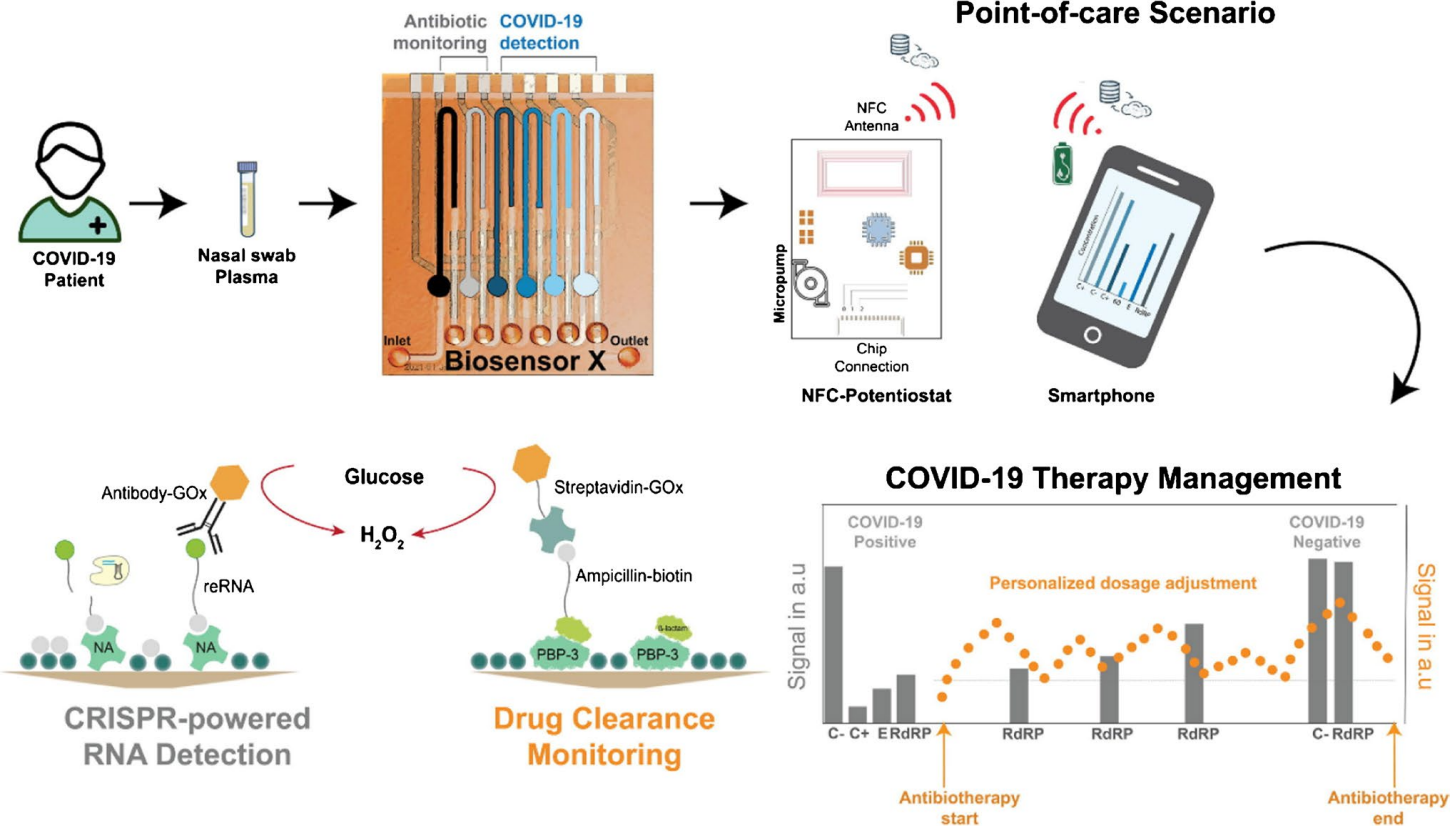
Microfluidic biosensing platform for the simultaneous determination of viral load and ß-lactam antibiotic in nasal swabs and serum samples, respectively, of COVID-19 infected patients. Reproduced from [70] with permission

Bioplatforms using PBPs exploit direct competitive formats in which the drug to be detected competes for binding to the PBP with a biotin-labeled analog which is further labeled with a commercial enzyme conjugate of streptavidin or avidin and glucose oxidase (GOx). Transduction is performed by amperometrically detecting H_2_O_2_ generated by enzymatic oxidation of glucose on a Pt electrode. These strategies were successfully applied in plasma and untreated blood samples. It is remarkable that one of these platforms is proposed for finger-prick testing [65], which allows samples to be processed more easily and quickly than traditional methods and can be safer and more convenient, particularly for certain types of patients such as pediatric, neonatal, and elderly patients for whom blood collection by venipuncture is difficult [65]. Another of these strategies has been implemented in a multiplexed microfluidic biosensing platform that allows the simultaneous determination of viral load (*E* and *RdRP* viral RNAs genes using CRISPR/Cas-powered assays) and ß-lactam antibiotic in nasal swabs and serum samples, respectively, of COVID-19 infected patients, thus allowing near real-time assessment of the therapy effectiveness on the treated infection (Fig. 6) [70].

**Fig. 7:**
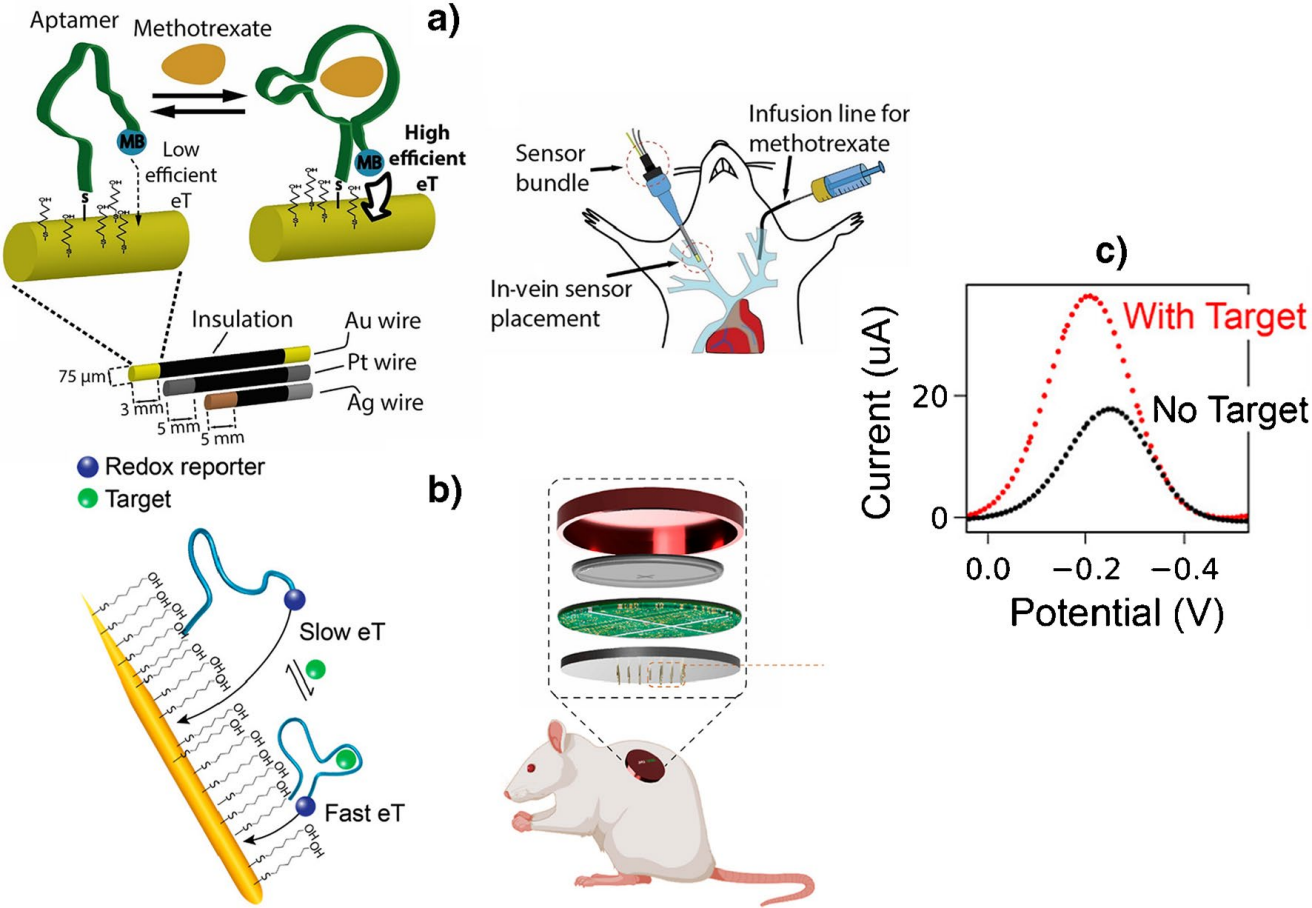
Electrochemical aptamer-based sensor involving a mixed monolayer of aptamers dually labeled with thiol and methylene blue (MB) and mercaptohexanol (MCH) self-assembled on gold wires a) or needles b) for *in vivo* continuous, real-time monitoring of methotrexate a) and rinotecan b) in live rats. c) In both cases electrochemical transduction is performed measuring the oxidation signal of MB by SWV. Reproduced from **a**) [34] and **b, c**) [33] with permission

Importantly, all the reported bioplatforms using aptamer switches have been shown to be able to provide real-time information in a single step, reagentless, and in a continuous way, even *in vivo* (most of them in jugular vein of live rats but also implanted in rat brain [67]). They exploited the use of antibiofouling SAMs to modify the electrode surface [29, 35] and chronoamperometric [69] or (mostly) voltammetric detection of the redox mediator to which the aptamer is conjugated. It is important to note that to eliminate the drift in electrochemical responses occurring *in vivo* deployments, these methods imply response treatments such as kinetic differential measurements (KDM) [30–34] (Fig. 7a) or the construction of a hypothetical baseline using an exponential equation which describes the readout drift pattern in the biofluid [62].

Aptamer switches-based bioplatforms have been used to develop calibration-free electrochemical biodevices [31, 35, 69], a closed-loop, feedback-controlled delivery system [68] and have recently been implemented in microneedle electrode arrays for *in vivo* determination in the ISF of a rodent (Fig. 7b) [33]. Aptamer switch-based bioplatforms are generalizable to the detection of a wide range of therapeutic agents as they are independent of the chemical or enzymatic reactivity of their targets [34].

We cannot end this section without highlighting the reagentless technology proposed for electrochemical biomolecular analysis using a double-stranded DNA, containing at its distal end an antibody that recognizes the target analyte, as well as a redox reporter that generates an electrochemical response, covalently immobilized on an electrode surface and called recently as molecular pendulum biosensors [71–73] with great potential for precision medicine and therapy. By applying a positive potential to the sensor interface, the negatively charged DNA pendulum is pulled towards the electrode surface and, when the target analyte binds to the antibody, the kinetics of the pendulum slows down [74]. This approach has demonstrated, in connection with chronoamperometric detection, applicability both to detect a wide range of proteins, related to stress, allergy, cardiovascular health, inflammation, COVID-19 infection and cancer, varying in charges, sizes and molecular weights, in various body fluids, including saliva, urine, tear fluid, blood, and sweat and to continuous and real-time *in vivo* measurements [71]. All these proteins were detected using the same reversible mechanism by simple substitution of the recognition antibody conjugated to the double-stranded DNA linker, indicating the versatility of this strategy to achieve user-friendliness without sacrificing sensitivity or universality, making it particularly attractive for multiplexing purposes.

## Precision nutrition

It is well known that an inadequate nutritional status can have profound long-term implications on physical and mental health. Conversely, an optimal nutritional intake, adapted or combined with specific nutrients such as vitamins (A, B6, B12, C, D3 and E) and minerals (zinc and iron), can boost the immune system, making the population less prone to certain diseases or helping to minimize their effects.

Currently the recommendations of physicians and dietitians continue to be the most widely used way to maintain optimal nutritional condition and prevent nutritional imbalances. These guidelines are based on population averages and overlook interpersonal variability, conditioned by genetic factors, lifestyle, microbiome composition, etc., which can greatly influence the desired results. This is the reason why recently precision nutrition, which aims to formulate precision dietary interventions and recommendations by tailoring nutrition according to the distinctive needs and/or sensitivities of each individual, has been gathering strength. This new approach is considered to have the potential to minimize, control and/or prevent the effects of food allergies and other chronic diseases such as obesity, diabetes, hypertension, celiac disease, cardiovascular diseases, etc., improving the expectations and quality of life of the population [4].

While strict control of nutrient/allergen content and food quality is the first step, close monitoring of biomarkers indicative of risky, adequate, or deficient nutrient intake is considered indispensable to ensure precision nutritional care.

Recent advances in digital nutrition technology, including mobile calorie counting apps and wearable motion tracking devices, lack the ability to monitor nutrition at the molecular level that requires the development of individualized nutritional strategies.

In this context, *in situ* monitoring of nutritional biomarkers in food and human body fluids using electrochemical bioplatforms is particularly attractive for providing personalized nutritional information [4]. Because of the great versatility of applications and developments, this type of biodevices have great interest for preventing nutritional imbalances and assessing compliance with nutrient intake and absorption dynamics. Moreover, their compatibility with multiplexed and/or multi-omics determinations make them as particularly suitable for these purposes considering the complexity and diversity of analytes involved in nutritional status and derived diseases [4].

However, despite the tremendous potential that electrochemical biosensors have unfolded in recent years in precision medicine and antibiotherapy, their incursion into tracking and guiding nutrition has emerged only recently and with a limited number of contributions.

Although it is possible to assist personalized nutrition by determining the molecular biomarker in the food to be ingested or directly in the biofluids, this review only discusses the bioplatforms used to determine molecular markers in body fluids. However, it is important to remark that electrochemical bioplatforms have been employed for the detection of allergens and food adulterations at the protein [75, 76] and genetic [77, 78] levels. Due to the higher integrity of DNA with respect to proteins during food processing, the development of electrochemical bioplatforms allowing the identification of genomic DNA from current allergens, such as mustard [78], and other targets derived from little explored plant and animal organelles, such as chloroplast and mitochondrial DNA [77], should be highlighted in this field. The flexibility showed by these biotools to detect allergenic targets, regardless of their omics level, origin (plant or animal) and type of organelle (nucleus, mitochondrion, or chloroplast), together with the versatility in format, design, and use, as well as the simplicity and capacity for in situ and multiplexed quantification allowed by current electrode substrates and electrochemical instrumentation, make electrochemical bioplatforms very promising tools for advancing precision nutrition.

Focusing on the bioplatforms reported for the determination of molecular markers related to personalized nutrition in biofluids, it is important to distinguish between those that have faced the determination of the molecular marker in the biofluid in a decentralized manner [79–84] or directly on the body (stimulated sweat) [82, 83]. Despite the variety of markers of interest contributing to individualized nutrition which include, among others, essential amino acids, vitamins, sugars, and specific immunoglobulins produced by the immune system against food allergens, most of these electrochemical bioplatforms reported for the determination of dietary biomarkers in bodily fluids interrogated vitamins. However, it is important to note that because of the dependence of the targets on nutrition, most of the works discussed in the precision medicine section, for example those reported for the determination of glucose, ketone bodies (KBs), ferritin, and cholesterol, can also find application in personalized nutrition.

An electrochemical immunosensor was reported by Kaur et al. [79] and an aptasensor was described by Wadhwa et al. [80] for the determination of vitamin D in real human serum samples. Both are label-free bioplatforms that exploited the immobilization of bioreceptors on electrode substrates modified with nanomaterials (AuPt NPs on APTES-FTO electrode for the immunosensor and GQD-Au hybrid particles on a gold microelectrode for the aptasensor) providing LOD values of 0.49 pg mL^-1^ and 0.84 ng mL^-1^ using DPV and impedimetric transduction, respectively.

**Fig. 8:**
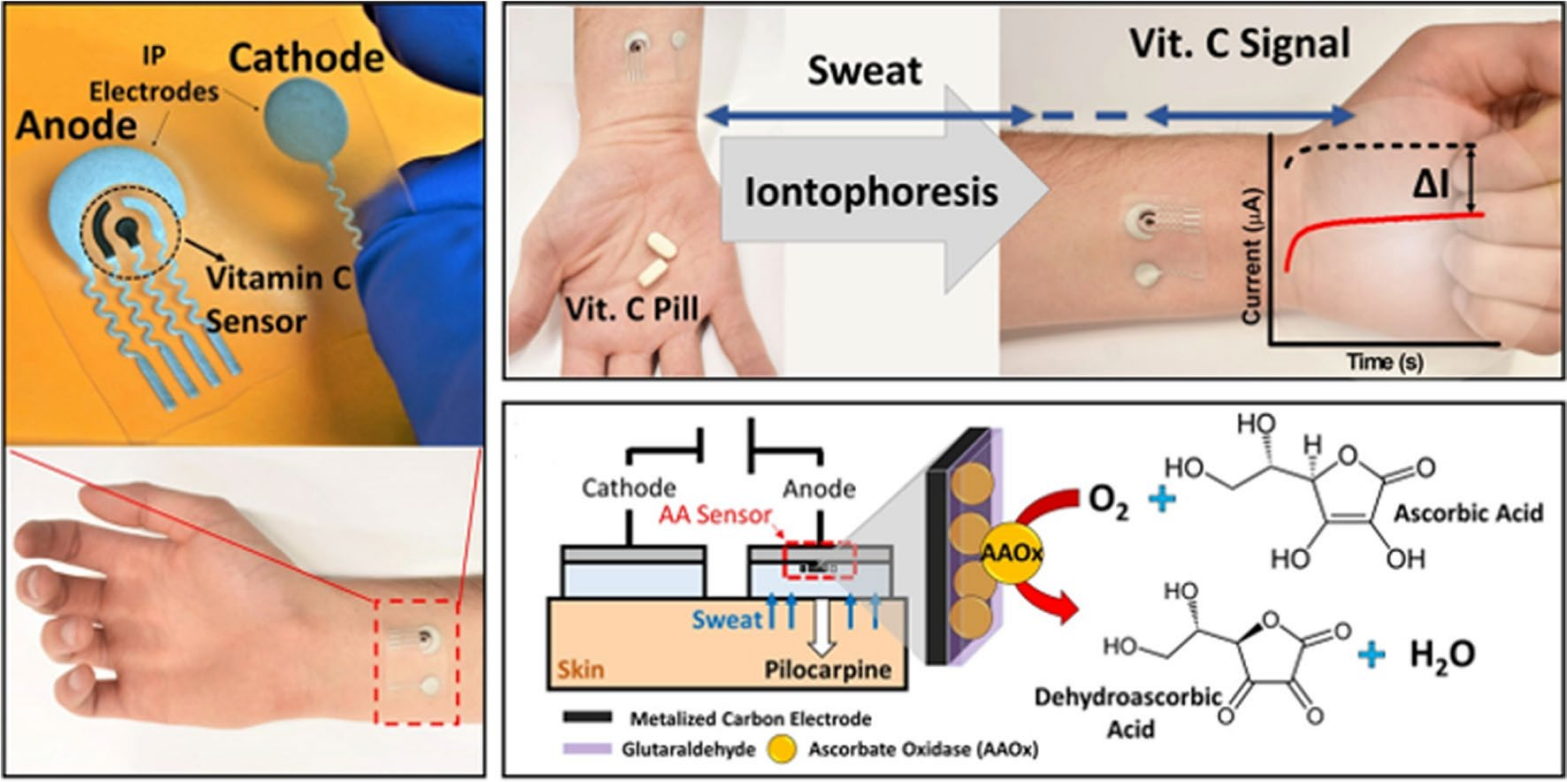
Tattoo patch type biosensor using the AAOx enzyme for the chronoamperometric determination of vitamin C in stimulated sweat. Reproduced from [82] with permission

Wang's team at UCSD tested in a pioneer way electrochemical chips for the decentralized determination of vitamins (vitamins C and D) in easily accessible biofluids (untreated saliva, tears, and sweat samples) [81, 82] by exploiting integrated catalytic, enzymatic or immunochemical sensing strategies. This team developed an ascorbate oxidase (AAOx) enzymatic biosensor and measured changes in the reduction current of the oxygen cosubstrate for the rapid *in vitro* determination of vitamin C in untreated raw saliva and tears or in stimulated sweat following pill or juice intake using disposable strip or skin-worn formats, respectively (Fig. 8) [82]. The same team reported the first example of a dual bioelectronic chip for tracking simultaneously vitamins C and D in saliva by integrating different (electrocatalytic and immunoassay) detection principles on a single chip platform. The bioelectronic sensor chip was able to perform rapid *in situ* detection of both vitamins in a 10-μL saliva sample in less than 25 minutes [81].

An illustrative example is the wearable electrochemical enzyme biosensor to selectively determine vitamin C in different biofluids (sweat, urine and blood) [83]. The biosensor used a gold electrode modified with gold nanostructures, LiClO_4_-doped conductive polymer, and a membrane immobilized with AAOx. The enzymatic oxidation of vitamin C was monitored by amperometry, and the biosensor was employed for tracking concentration changes in sweat and urine profile after vitamin C intake and for longitudinal studies of the correlation of sweat and urine vitamin C with blood concentrations.

Disposable electrochemical bioplatforms have been developed for the reliable determination of food allergen-specific IgE and IgG4 in serum [84]. The strategy relied on the implementation of indirect immunoassays on the surface of magnetic microsupports captured on the surface of disposable electrode surfaces to perform amperometric transduction and was tuned for the determination of ovalbumin-specific immunoglobulins in children allergic to eggs. The bioplatform allowed the determination of both isotypes without matrix effect after a serum dilution (50 or 1000-fold depending on the Ig class) in an optimal time of 90 min that can be reduced to 10 min if necessary.

## Remarkable notes, challenges, and personal prospects

The great advances occurred in different disciplines (electrode substrate and electronics fabrication, miniaturization, bioengineering, etc...) in recent years and their perfect combination have allowed the development of cutting-edge electroanalytical biotools, with the ability to monitor in a sensitive, selective, simple, continuous, and almost real time way, molecular markers directly in the biological fluids extracted or secreted either naturally or stimulated from the body or circulating in it. These advances make us to believe that precision medicine, therapy, and nutrition is not a utopia and that if we continue to advance together in different areas, it is possible that in a near future our phones or smart watches will have these biotools integrated to ensure our welfare.

Although the advances occurred in recent years seem like giant steps, reaching the goal is neither close nor easy. However, the unquestionable individual and social benefits of doing so and the empowerment of the spirits when seeing what has been advanced in such a short time are the best fuels to stay on track and achieve precision in our lives.

Electrochemical biotools have surprised by using edible electrode materials or cell membranes as bioreceptors and providing reliable information of an individual or multiple nature in near real time, without the need for calibration and continuously, even moving the analysis in the laboratory to our body with wearable or implantable formats.

In recent years we have been privileged witnesses to the application of electrochemical biosensor platforms for the analysis of naturally occurring sweat on a fingertip, of biological fluids with denaturing pH values such as gastrointestinal pH or of two analytes in a single microliter of untreated biofluid in less than 30 minutes and of others that have emerged in a record time to address the unexpected COVID-19 pandemic. Among the latter, are the biosensors developed to evaluate different key aspects of the disease such as viral load, immune responses, and the severity with which the infection is experienced, as well as the “quantification” of natural and/or acquired immunity after infection and/or vaccination processes, and the identification of the variant responsible for the infection and the evaluation of the degree of protection against any variant that may arise.

The versatility of these bioplatforms seems to have no limit and they have, for example, integrated in the same chip catalytic and affinity bioassays or immunoassays with different formats and enzymatic tracers. Also, they have been successfully coupled with acoustic sensors to allow the simultaneous determination of hemodynamic and metabolic biomarkers.

The ability of using antigens of different nature, identified by directed proteomics including circulating or exosomal antigens, peptides, proteoforms, etc., and produced by technologies such as HaloTag or phage display, allows the identification and the diagnostic value assessment of new molecular signatures comprising 6-15 AAbs for the early and minimally invasive detection of diseases as cancer and Alzheimer.

No less relevant are considered the opportunities provided by electrochemical biodevices for improving TDM using finger-prick testing or implanted in live rats in connection with aptamer switches or bacterial antibiotic-binding proteins, or for the advances in the still less explored precision nutrition.

So far, the use of electrochemical bioplatforms in precision nutrition is mainly focused on the determination of vitamins in extracted or secreted body fluids. This may be justified because many biomarkers such as glucose, KBs, ferritin, and cholesterol are of interest in both medicine and precision nutrition, and they have been sold by the former, probably more fashionable field. Nevertheless, it does not question their potential to revolutionize also individualized nutrition.

As a result of these relentless advances, a multitude of challenges are blossoming, among which we can highlight the need for these devices to demonstrate their usefulness outside the research laboratories, the need for addressing the correlation between the molecular markers concentrations provided by these biodevices in fluids such as saliva and sweat with the concentrations in blood and the need for validation with much larger cohorts of individuals in different environments and by different users, which goes through the involvement of several partners (e.g. industry, hospitals, and government body agencies) and specific workflows and infrastructures. So far, the featured bioplatforms have provided data on only a limited realistic number of samples (which is required after development in the course of academic proof of concept and for publication in peer-reviewed scientific journals) and with this it is assumed that the theory works for the proposed application, and this is too much to assume. The overcoming of these challenges will be decisive in awakening in producers, users, and society, in general, the interest, motivation, and conviction necessary to continue empowering these devices and facilitating their translation into daily lives.

Combining the advances, challenges to be faced and our experience, we envision a challenging and exciting roadmap for these cutting-edge electroanalytical biotools, working in close collaboration with, among others, electronic engineers, biostatisticians, biotechnologists, clinicians, and nutritionists.

Precision medicine must exploit the panoply of available bioreceptors (antibodies, aptamers, peptides, etc.), in addition to enzymes, for the detection of molecular markers other than metabolic markers and belonging to different omics levels of both proven and as yet unproven clinical relevance, to identify molecular signatures that contribute to improve the resolution of each patient’s snapshot. Due to the infancy and complexity of the subject matter, it is not reasonable at this time to rely on specific biofluids or to demand particular biotools operation, or performance characteristics beyond simplicity, reliability, versatility, and affordability. All these aspects will have to be agreed upon on a case-by-case basis, considering multiple factors such as application, environment, user profile, etc. It is precisely the versatility of the electrochemical bioplatforms design what allows the menu to be left open to the consumer's taste.

To impart greater precision to the therapy it would be interesting to address with these bioplatforms the joint detection of the drug and other markers related to the efficiency and/or toxicity of the therapy (markers of infection, inflammation, renal function, etc.) in order to “hit the bull’s eye” with the most appropriate therapy for each patient.

Nutrition, like precision medicine, cries out for these bioplatforms to enter it hand by hand with other bioreceptors and to determine markers other than vitamins, sugars and fats, such as those related to food allergies and intolerances (e.g. specific immunoglobulins).

It is important to note that, although in this review article, and for simplicity, they have been discussed independently, the interconnection between nutrition, medicine, and precision therapy, which is exemplified very well in the consumption of foods with components for which we are particularly sensitized, would require studying and implementing them together with this type of biodevices.

With the development of these new biotools, and the access to all the expected individualized, objective, and quantitative information provided in a sustainable and equal manner, we no longer have excuse to get involved in our self-care by doing everything in our power to live longer and better and to ensure a rational use of limited social resources.

Let’s keep dreaming that in not too many years we will have the possibility of choosing to implant intelligent microneedle patches, like the currently used by a large part of the population with DM, that work for precision nutrition and medicine, so that we will all live longer and better.

## Data Availability

Not applicable.
